# Multi-locus identification of *Psilocybe cubensis* by high-resolution melting (HRM)

**DOI:** 10.1080/20961790.2021.1875580

**Published:** 2021-04-13

**Authors:** Xiaochun Zhang, Huan Yu, Ziwei Wang, Qi Yang, Ruocheng Xia, Yiling Qu, Ruiyang Tao, Yan Shi, Ping Xiang, Suhua Zhang, Chengtao Li

**Affiliations:** a Department of Forensic Science, Medical School of Soochow University, Suzhou, China; b Shanghai Key Laboratory of Forensic Medicine, Shanghai Forensic Service Platform, Academy of Forensic Sciences, Ministry of Justice, Shanghai, China; c Department of Forensic Medicine, School of Basic Medical Science, Wenzhou Medical University, Wenzhou, China; d Institute of Forensic Medicine, West China School of Basic Medical Sciences & Forensic Medicine, Sichuan University, Chengdu, China

**Keywords:** Forensic sciences, forensic genetics, polymerase chain reaction (PCR), high-resolution melting (HRM), *Psilocybe cubensis*, multi-locus, species-specific

## Abstract

Hallucinogenic mushroom is a kind of toxic strain containing psychoactive tryptamine substances such as psilocybin, psilocin and ibotenic acid, etc. The mushrooms containing hallucinogenic components are various, widely distributed and lack of standard to define, which made a great challenge to identification. Traditional identification methods, such as morphology and toxicology analysis, showed shortcomings in old or processed samples, while the DNA-based identification of hallucinogenic mushrooms would allow to identify these samples due to the stability of DNA. In this paper, four primer sets are designed to target *Psilocybe cubensis* DNA for increasing resolution of present identification method, and the target markers include largest subunit of RNA polymerase II (marked as PC-R1), psilocybin-related phosphotransferase gene (marked as PC-PT), glyceraldehyde 3-phosphate dehydrogenase (marked as PC-3) and translation EF1α (marked as PC-EF). Real-time PCR with high-resolution melting (HRM) assay were used for the differentiation of the fragments amplified by these primer sets, which were tested for specificity, reproducibility, sensitivity, mixture analysis and multiplex PCR. It was shown that the melting temperatures of PC-R1, PC-PT, PC-3 and PC-EF of *P. cubensis* were (87.93 ± 0.12) °C, (82.21 ± 0.14) °C, (79.72 ± 0.12) °C and (80.11 ± 0.19) °C in our kinds of independent experiments. Significant HRM characteristic can be shown with a low concentration of 62.5 pg/µL DNA sample, and *P. cubensis* could be detected in mixtures with *Homo sapiens* or *Cannabis sativa*. In summary, the method of HRM analysis can quickly and specifically distinguish *P. cubensis* from other species, which could be utilized for forensic science, medical diagnosis and drug trafficking cases.

Supplemental data for this article are available online at https://doi.org/10.1080/20961790.2021.1875580.

## Introduction

*Psilocybe cubensis*, *Psilocybe merdaria*, *Panaeolus antillarum* and other related species are known as “sacred mushrooms” or “hallucinogenic mushrooms”, which have been used for hundreds of years in certain religious rituals of indigenous Mexicans [1]. The neurotoxin with hallucinogenic effects contained by these mushrooms is psilocybin, which mainly exists in four genera: *Psilocybe*, *Panaeolus*, *Conocybe* and *Gymnopilus* [2–4]. Since the 1970s, mushrooms containing these hallucinogens have been commonly eaten by young people in the US, Canada, the UK, Germany and other European countries for recreational purposes [5]. However, a long-term obsession with such substances will cause neurotoxicity, and the out-of-control of behaviours will easily lead to the occurrence of criminal cases [6]. On account of the psilocybin-induced hallucinations and intensified emotions, the non-medical administration of psilocybin might also induce dangerous behaviours or even suicides [7, 8]. To prevent such drug-related deaths and investigate drug trafficking cases, the detection and identification of hallucinogenic mushrooms is absolutely needed for emergency medical treatment and criminal proceeding.

Traditional species identification of hallucinogenic mushrooms has gone through five stages: morphology, cytology, bio-chemistry, immunology and molecular biology [9–11]. However, different materials are subject to different environmental factors, and the degree of their changes in denaturation, degradation and corruption could be unpredictable, which brings difficulty to the identification work. As a novel species classification technology, DNA-based identification provides a new direction for taxonomic study and has a broad application prospect in biology, medicine, food hygiene, forensic medicine and other fields [12–14]. For example, internal transcribed spacer (ITS) and its fragments ITS1 and ITS2 are highly polymorphic between species and has been characterized as universal and effective DNA barcoding [13, 14], while Nugent and Saville [15] found that nLSU rRNA (28S) has better identification effect than ITS1 on hallucinogen containing and non-hallucinogen containing mushrooms. Besides these barcodes, protein coding-gene *RPB1* has been used in the classification and identification of Inocybe and Agaricales, and *RPB2* has been used in combination with LSU and SSU to study the phylogeny of Ascomycota [16, 17]. Although more and more regions have been excavated for distinguishing species, there is still no complete identification strategy designed for *P. cubensis*. The ITS region was widely used for the discrimination of *P. cubensis*, while its high universality resulted in unstable species specificity. Other DNA markers have not been studied on *P. cubensis* comprehensively. The purpose of this study was to select some markers unique for *P. cubensis* and make a combination of them, designing an identification strategy specifically for *P. cubensis* in forensic science.

The high-resolution melting (HRM) analysis is a method used for rapid analysis of DNA sequence varia­tions within PCR amplicons, which displays specific HRM curve parameters and melting temperatures (*T*m) by monitoring fluorescence changes [18–21]. This technology could provide a detailed analysis of nucleotide variations between amplicons acquired from different species. For example, the real-time PCR followed by HRM assay has been demonstrated for identifying *P. cubensis*, *Cannabis sativa*, *Datura stramonium* and *Merremia tuberosa* in a single multiplex PCR reaction [2, 19, 20]. In this study, four primer sets were designed to target *P. cubensis*, and real-time PCR HRM was performed to analyze the characteristic of each marker, including specificity, reproducibility, sensitivity and identification performance in mixtures and multiplex PCR. The primers were tested across 22 species, including *P. cubensis*, related mushrooms or plants and *Homo sapiens*. The results showed the method can clearly distinguish *P. cubensis* from other species, providing a reliable strategy for forensic identification of *P. cubensis*.

## Materials and methods

### Samples collection

Multiple species materials were recruited for following experimental analysis. The hallucinogenic mushrooms of *P. cubensis*, *P. merdaria*, *Panaeolus papilionaceus* and hallucinogenic plant of *C. sativa* were almost gathered from forensic laboratories. Other wild mushrooms and plant materials used for analysis were all collected outdoors, which from Guangxi, Yunnan, Jiangsu, Shanghai and other places in China. All species have been initially identified by mycological experts according to morphological characteristics, and the ITS regions of all mushrooms were then sequenced and aligned with sequences in National Center for Biotechnology Information (NCBI) GenBank using a nucleotide BLAST search https://blast.ncbi.nlm.nih.gov/Blast.cgi to confirm their species. The material symbols, scientific name, family, source and the accession number were summarized in Table 1.

**Table 1.** *Psilocybe cubensis* and other species used in this study.

Symbol	Scientific name	Family	Source	Hallucinogenic	Accession No (identity)
Pc	*Psilocybe cubensis*	Strophariaceae	Laboratory	Yes	MN893872 (100%)
Pm	*Psilocybe merdaria*	Strophariaceae	Laboratory	Yes	AB158636 (100%)
Sr	*Stropharia rugosoannulata*	Strophariaceae	Shanghai	No	MN893872 (100%)
Ap	*Amanita parvipantherina*	Amanitaceae	Guangxi	Yes	MH508498 (100%)
As	*Amanita subglobosa*	Amanitaceae	Laboratory	Yes	MK388157 (100%)
Bt	*Bolbitius titubans*	Bolbitiaceae	Yunnan	Yes	KR425521 (99.50%)
La	*Lanmaoa asiatica*	Boletaceae	Yunnan	No	MG030477 (99.87%)
Ig	*Inocybe geophylla*	Cortinariaceae	Guangxi	Yes	FN550916 (100%)
Gp	*Gymnopilus penetrans*	Cortinariaceae	Hunan	Yes	KT368685 (100%)
Il	*Inocybe lacera*	Cortinariaceae	Yunnan	Yes	FJ553061 (99.08%)
In	*Inocybe nitidiuscula*	Cortinariaceae	Yunnan	Yes	HQ604086 (100%)
Os	*Oudemansiella submucida*	Marasmiaceae	Guangxi	No	AY804290 (99.85%)
Pp	*Panaeolus papilionaceus*	Psathyrellaceae	Laboratory	Yes	MK439503 (99.18%)
Pf	*Psathyrella fimetaria*	Psathyrellaceae	Guangxi	No	MH860432 (100%)
Ca	*Coprinopsis atramentaria*	Psathyrellaceae	Jiangsu	No	MN258631 (99.56%)
Pa	*Panaeolus antillarum*	Psathyrellaceae	Hunan	Yes	MF497586 (100%)
Ps	*Panaeolus sphinctrinus*	Psathyrellaceae	Laboratory	Yes	KY559331 (99.68%)
Mh	*Mycena haematopus*	Tricholomataceae	Hunan	No	LT716053 (100%)
Cp	*Clitocybe phyllophila*	Tricholomataceae	Yunnan	Yes	MK966602 (99.80%)
Cc	*Clitopilus crispus*	Tricholomataceae	Yunnan	No	MN061316 (100%)
Cs	*Cannabis sativa*	Moraceae	Laboratory	Yes	­­–
Hs	*Homo sapiens*	Hominidae	Laboratory	No	–

### Genomic DNA extraction

The pileus on fruiting body of the mushrooms was thoroughly ground, and the genomic DNA of samples was extracted using the DNeasy Plant Pro Kit according to the manufacturer’s protocol (Qiagen, Hilden, Germany). All templates were quantified with NanoDrop 2000 spectrophotometer (NanoDrop Technologies Inc., Wilmington, DE, USA), analyzed by NanoDrop 2.4.7c software (NanoDrop Technologies Inc.) according to the manufacturer’s recommendations and diluted to 1.0 ng/µL for real-time PCR HRM analysis.

### Markers selection and PCR primers design

Four sequences related to *P. cubensis* were downloaded from the NCBI database, including the largest subunit of RNA polymerase II (*RPB1*), psilocybin-related phosphotransferase gene, glyceraldehyde 3-phosphate dehydrogenase gene and translation EF1α (*tEF1α*). The software Primer Premier 5.0 (Premier Biosoft International, San Francisco, CA, USA) was utilized to design PCR primers for the selected regions. The primer length was required to be 20–30 bp with a *T*_m _of approximately 55 °C and the GC content varying between 40% and 60%. If possible, the hairpins, self- and heterodimers should also be avoided. Primers were synthesized at Sangon Biotech (Shanghai, China), quantified using NanoDrop 2000 spectrophotometer and diluted to 10 µmol/L. The primer symbols and detailed information were listed in Table 2.

**Table 2.** Primers for amplifying DNA barcoding of *Psilocybe cubensis* and other species.

Symbol	Primer sequences (5′ → 3′)	NCBI accession number (range)	Length (bp)	Gene	*T*_m_ (°C)
PC-R1	F: CTCTACTCGTTTCGCACCC	KC669344	246	Largest subunit of RNA polymerase II (*RPB1*)	55.90
	R: CGCACTCCTCGTTCAGC				57.07
PC-PT	F: ATCGGGAGGTTCTGGG	MG548657	145	Psilocybin-related phosphotransferase	54.01
	R: CGCAAGTGGCGGTTT				53.80
PC-3	F: CGCTGGTGCTGAATACG	KM273235	182	Glyceraldehyde 3-phosphate dehydrogenase	54.06
	R: CGGAAGCCCTGGAAAG				53.45
PC-EF	F: TTCATCAAGAAGGTCGGTTAC	HF912338	67	Translation EF1α (*tEF1α*)	51.75
	R: TCTCCGTGCCATCCAG				54.63

### PCR HRM reaction master mixes and analysis setting

In the study, Type-it HRM PCR Kit (Qiagen) was used in real-time PCR reaction. Eva GreenTM intercalating dye was selected to monitor the accumulation of amplified product during the process of real-time PCR and HRM. The excitation and emission spectra of EvaGreenTM were very close to those of fluorescein, with the advantage of no PCR inhibition, stronger combinatorial ability and higher GC analysis accuracy [22]. Standard amounts in the mixture totalled 25 µL consisting of 12.5 µL of 2× HRM PCR Master Mix, 9.75 µL of RNase-Free Water, 1.75 µL of primers mix (10 µmol/L) and 1 µL of template DNA (1 ng/µL).

Rotor-Gene Q Serious (Qiagen) was utilized in our experiment. The standard thermocycling/melting conditions were: initial hold at 95 °C for 5 min; then cycling denaturation at 95 °C for 10 s, annealing at 55 °C for 30 s, and extension at 72 °C for 10 s. This cycle was repeated 45 times. Next, amplicons were melted to a ramp from 65 °C to 95 °C, rising by increments of 0.1 °C and waiting 2 s for each step. Melt temperature peaks were visualized under Green through the Rotor-Gene Q Serious software V2.1.0 by computing the negative first derivative of collected fluorescence values (−*dF*/*dT*), which were plotted in their inverse format.

### Specificity and accuracy studies

Four primer sets designed for the target markers of *P. cubensis* were tested across 22 species with real-time PCR HRM analysis, including 20 mushrooms, *C. sativa* and human. The master mix parameters were performed for all specificity reactions and the melt conditions remained standard with each reaction. For quality control, one of *P. cubensis* samples was selected as positive control and examined in each of our HRM test. The negative control sample using the same amount of RNase-Free Water was also analyzed under the same conditions. Moreover, the four PCR amplicons of *P. cubensis* were analyzed by Sanger sequencing, the results of which were compared with the sequences available in GenBank in order to validate accuracy of the HRM method.

### Sensitivity and reproducibility studies

To evaluate the robustness of the HRM method, a serial of five DNA dilution of *P. cubensis* from 1.0 ng/µL to 62.5 pg/µL (i.e. 1 000, 500, 250, 125 and 62.5 pg/µL) were prepared for analyzing the sensitivity of the method. The *dF*/*dT* information of each reaction was recorded for the following assessment. Furthermore, five unrelated and independent *P. cubensis* samples obtained from forensic laboratory were examined and labelled as a–e. Blind trials on all samples were also performed in another accredited laboratory for reproducibility studies.

### Mixture analysis

In some cases, it is often necessary to test the gastric contents or the mixture of various drugs. The four primer sets were therefore applied to analyze DNA of *P. cubensis* mixed with *H. sapiens* or *C. sativa*. The DNA mixes tested in our study contained a total of 1 ng DNA, which consist of 0.5 µL of *P. cubensis* DNA (1 ng/µL) and 0.5 µL of DNA of *H. sapiens* or *C. sativa* (1 ng/µL). The reaction conditions and settings remain unchanged.

### Multiplex PCR HRM

We next sought to improve the identification efficiency by means of simultaneous detection of multiple DNA markers. The primer sets, which amplified PCR fragments with *T*_m_ differing by more than 2 °C, would be selected in same groups. Each group of primers was applied to perform a single multiplex PCR HRM on *P. cubensis*. The total reaction volume was still 25 µL, and the volume of RNase-Free Water was reduced and replaced by the increased volume of the selected primers (1.75 µL) so that multiple primer sets could be contained in a single PCR reaction mix.

## Results

### Specificity studies

**Figure 1.**
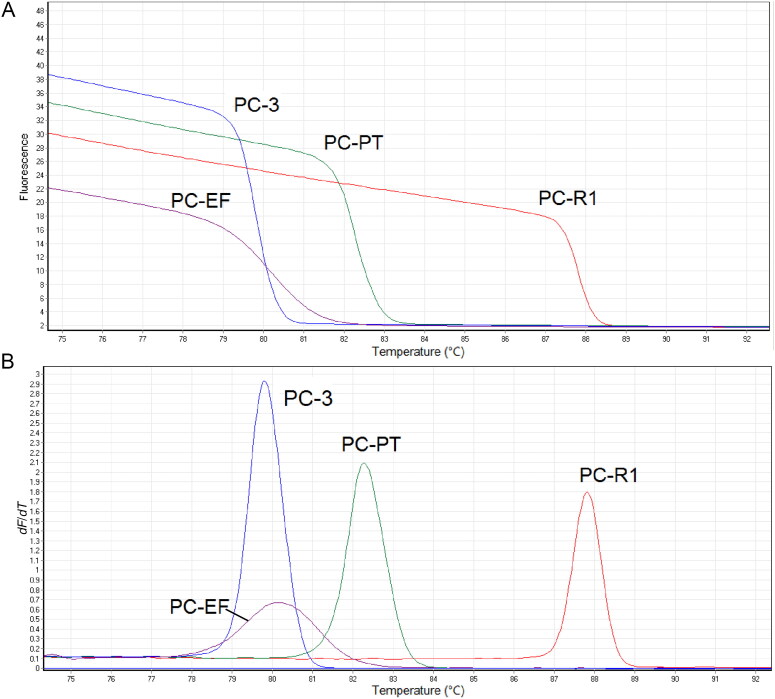
High-resolution melting (HRM) analysis of four DNA markers on *Psilocybe cubensis*. (A) Melting curves plot; (B) normalized melting peaks. RNA polymerase II: PC-R1; psilocybin-related phosphotransferase gene: PC-PT; glyceraldehyde 3-phosphate dehydrogenase: PC-3; translation EF1α: PC-EF.

We first analyzed the performance of four DNA markers on *P. cubensis* through HRM method. The characteristic HRM curves observed for *P. cubensis*, including normalized melting curve and derivative melt curve, were shown in Figure 1. All loci provided successful melting profiles for *P. cubensis*, and the observed *T*_m_ of RNA polymerase II (PC-R1), psilocybin-related phosphotransferase gene (PC-PT), glyceraldehyde 3-phosphate dehydrogenase (PC-3), translation EF1α (PC-EF) of *P. cubensis* were 87.93 °C, 82.21 °C, 79.72 °C and 80.11 °C, respectively. The PCR amplicons of *P. cubensis* were then analyzed by Sanger sequencing. The sequencing data are perfectly consistent with the sequences of four markers of *P. cubensis* available from Genebank, demonstrating that the designed primers are priming at the target regions.

Primers used above were then tested across 22 species to compare the specificity of each primer set, including *P. cubensis*, 19 related mushrooms, *C. sativa* and *H. sapiens* (Supplementary Figure S1). The observed *T*_m_ for all species were summarized in Table 3. Primer set PC-R1 and PC-EF successfully amplified 14 and 11 species, respectively, indicating the relatively low specificity of two primer sets. Primer set PC-PT successfully amplified eight species, and primer set PC-3 successfully amplified only five species. No amplification was observed from *Psathyrella fimetaria*, *Coprinopsis atramentaria*, *C. sativa* and *H. sapiens*, while four markers were all amplified successfully in *P. cubensis* and *Stropharia rugosoannulata*. The reason might be that they both belong to the family of Strophariaceae and are closely related. Despite this limitation, there are significant differences in *T*m values of PC-3 and PC-EF between *P. cubensis* and *S. rugosoannulata*, which provide enough evidences for discrimination of these two species. No analysis result was shown on the negative control samples (Supplementary Figure S2).

**Figure 2.**
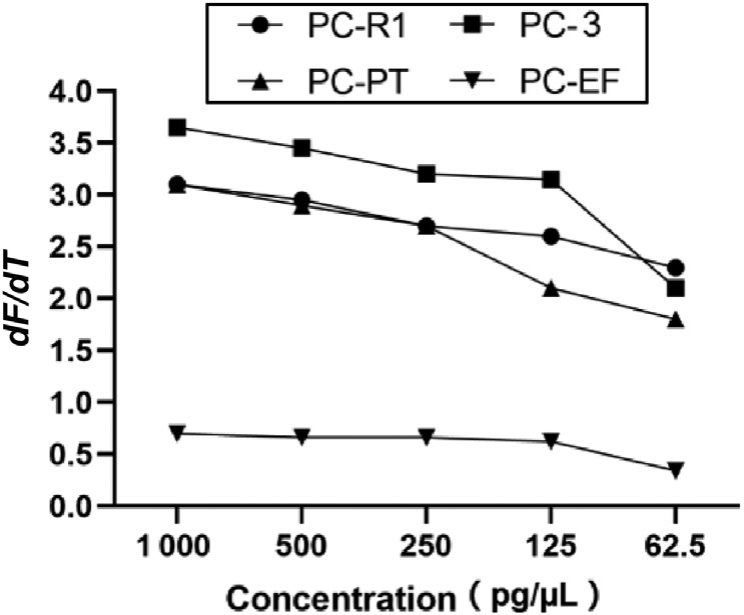
The *dF*/*dT* values of different PCR products observed at 1 000, 500, 250, 125 and 62.5 pg/µL DNA for *Psilocybe cubensis*.

**Table 3.** Results of melting temperatures (*T*_m_) of high-resolution melting (HRM) for all included species.

Species	*T*_m_ of PC-R1 (°C)	*T*_m_ of PC-PT (°C)	*T*_m_ of PC-3 (°C)	*T*_m_ of PC-EF (°C)
*Psilocybe cubensis*	87.93 ± 0.12	82.21 ± 0.14	79.72 ± 0.12	80.11 ± 0.19
*Psilocybe merdaria*	86.90 ± 0.20	87.68 ± 0.27	–	81.52 ± 0.24
*Stropharia rugosoannulata*	88.00 ± 0.22	81.53 ± 0.18	83.98 ± 0.05	81.55 ± 0.25
*Amanita parvipantherina*	86.23 ± 0.20	–	–	79.92 ± 0.26
*Amanita subglobosa*	85.70 ± 0.22	–	–	–
*Bolbitius titubans*	87.65 ± 0.27	–	–	–
*Lanmaoa asiatica*	–	–	–	85.01 ± 0.09
*Inocybe geophylla*	88.80 ± 0.28	85.05 ± 0.13	–	–
*Gymnopilus penetrans*	88.95 ± 0.18	80.18 ± 0.26	83.48 ± 0.11	–
*Inocybe lacera*	89.15 ± 0.24	–	–	–
*Inocybe nitidiuscula*	88.57 ± 0.13	85.18 ± 0.03	–	–
*Oudemansiella submucida*	–	–	–	80.60 ± 0.23
*Panaeolus papilionaceus*	87.02 ± 0.18	87.72 ± 0.23	–	74.40 ± 0.05
*Psathyrella fimetaria*	–	–	–	–
*Coprinopsis atramentaria*	–	–	–	–
*Panaeolus antillarum*	85.55 ± 0.26	–	79.93 ± 0.12	80.21 ± 0.23
*Panaeolus sphinctrinus*	86.82 ± 0.22	–	–	79.53 ± 0.12
*Mycena haematopus*	–	–	–	80.02 ± 0.15
*Clitocybe phyllophila*	–	–	–	86.05 ± 0.07
*Clitopilus crispus*	87.95 ± 0.20	82.86 ± 0.25	83.61 ± 0.07	–
*Cannabis sativa*	–	–	–	–
*Homo sapiens*	–	–	–	–

### Sensitivity, reproducibility and concordance studies

The identification performance of four primer sets was validated over the course of multiple independent experiment runs and samples varying in DNA concentration. Firstly, a serial of DNA concentration dilutions (1 000, 500, 250, 125 and 62.5 pg/µL) of *P. cubensis* were used to evaluate the sensitivity of the PCR HRM method. As shown in Supplementary Figure S3 and Figure 2 complete melting peaks can be obtained in all dilutions. The *dF*/*dT* values decreased when the concentration of DNA sample ranged from 1 000 down to 125 pg/µL, with the *dF*/*dT* dropped even faster when the DNA concentration were down to 62.5 pg/µL.

**Figure 3.**
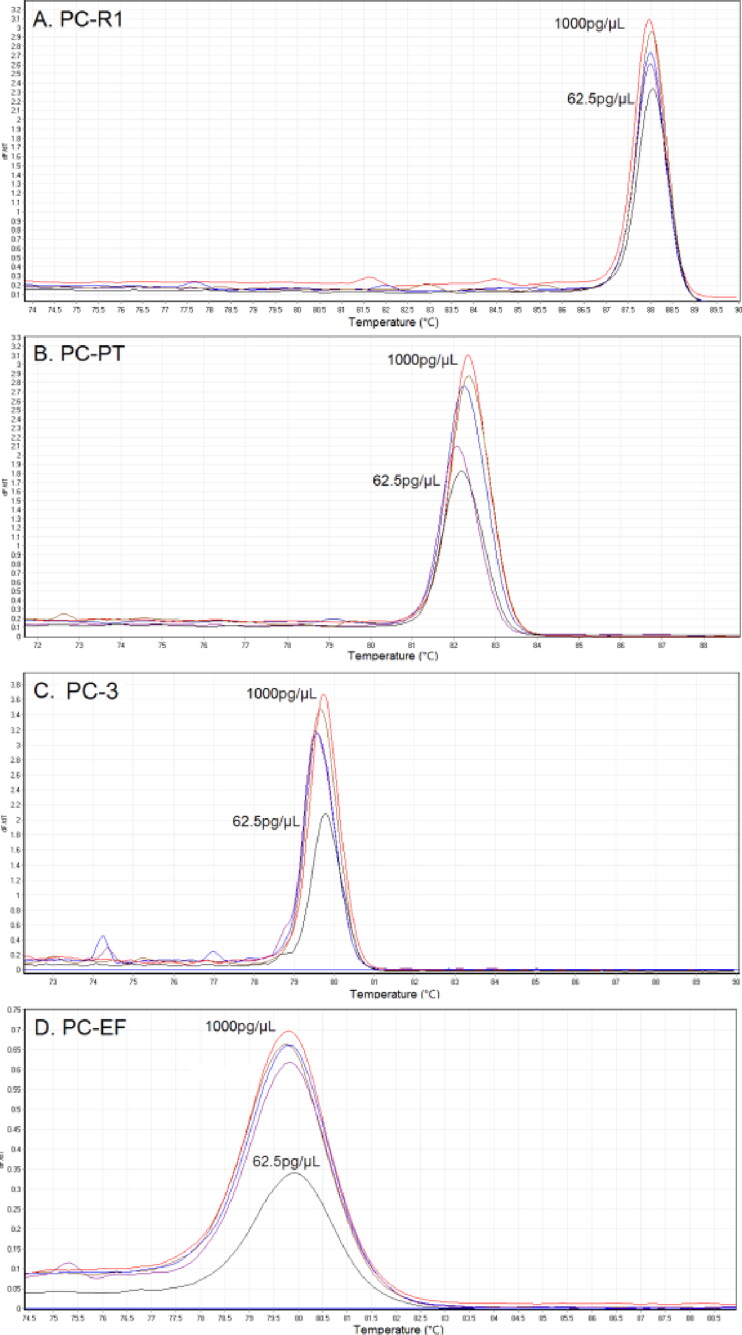
Dilution of *Psilocybe cubensis* with primers (a) PC-R1, (b) PC-PT, (c) PC-3 and (d) PC-EF at concentrations of 1 000, 500, 250, 125 and 62.5 pg/µL using Eva Green™ intercalating dye.

**Figure 2.**
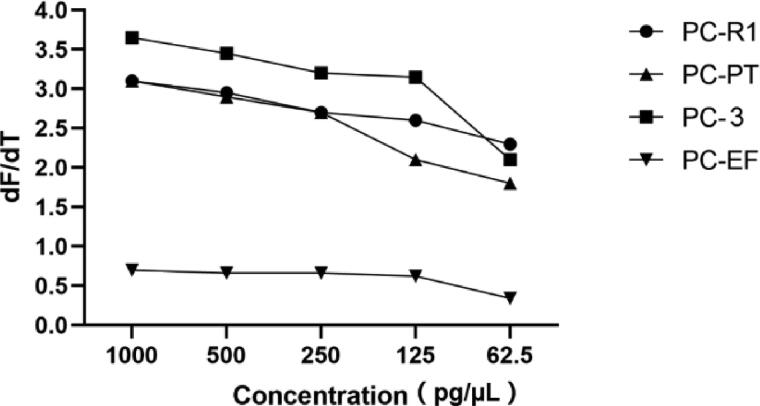
The *dF*/*dT* values of different PCR products observed at 1 000, 500, 250, 125 and 62.5 pg/µL DNA for *Psilocybe cubensis*.

To assess reproducibility of our assay, all samples were amplified in another laboratory and tested by HRM, obtaining consistent results with ours. Five unrelated and independent *P. cubensis* samples were also analyzed by HRM, aiming to evaluate the concordance of HRM method (Supplementary Figure S4). The observed *T*_m_ of target regions PC-R1, PC-PT, PC-3, and PC-EF were (87.93±0.12) °C, (82.21±0.14) °C, (79.72±0.12) °C, and (80.11±0.19 °C), respectively, which showed high stability of our method.

**Figure 5.**
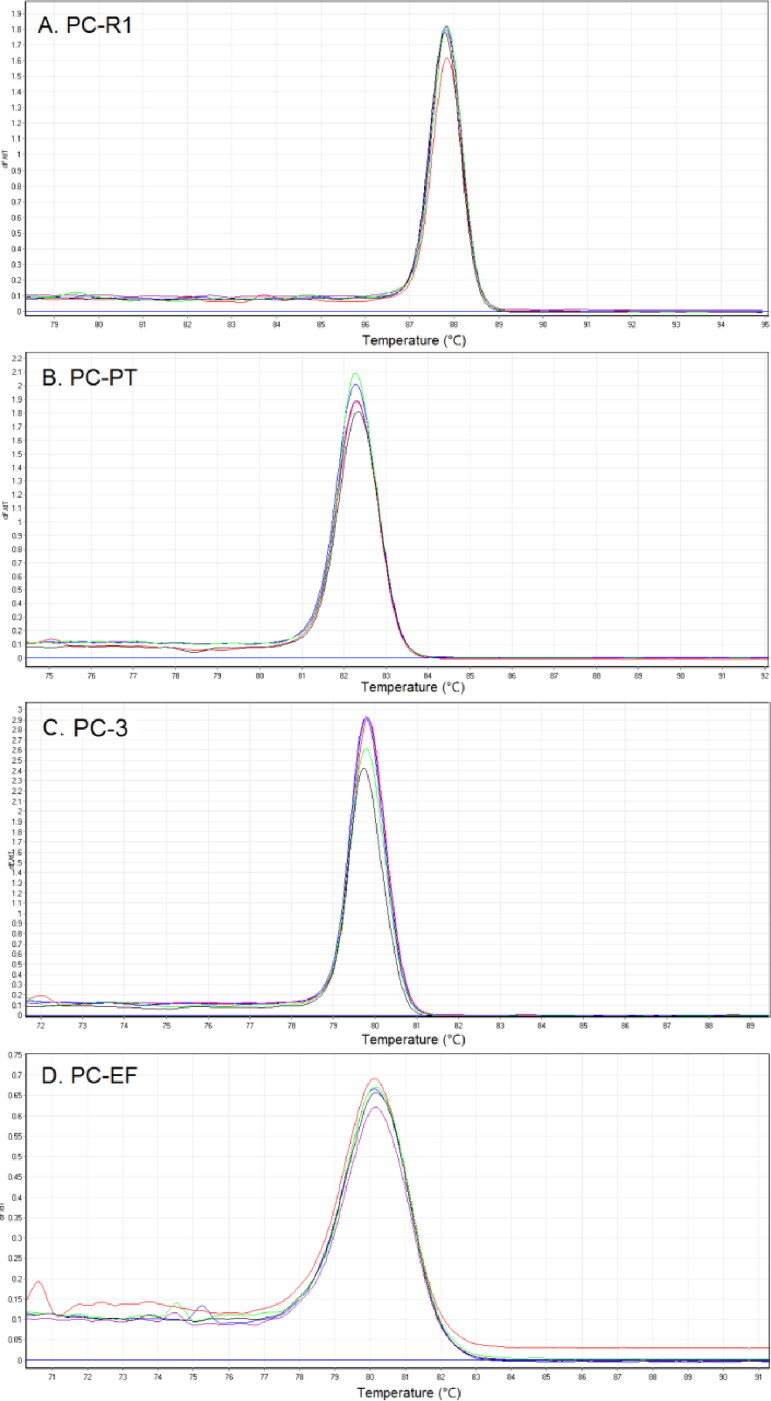
Reproducibility of primer set (a) PC-R1, (b) PC-PT, (c) PC-3 and (d) PC-EF on *Psilocybe cubensis*. a (red), b (green), c (blue), d (black) and e (purple).

### Mixtures analysis

In the mixtures, four DNA target regions of *P. cubensis* were all amplified perfectly. The HRM curves and *T*_m_ values of two mixtures almost overlapped with the control of single *P. cubensis* or only fluctuated between the range of the average and the standard deviation (Figure 3). The observed *T*_m_ of DNA markers in mixtures containing *H. sapiens* were 87.89 °C (PC-R1), 82.43 °C (PC-PT), 79.85 °C (PC-3) and 80.12 °C (PC-EF), while the observed *T*_m_ in mixtures containing *C. sativa* were 87.85 °C (PC-R1), 82.22 °C (PC-PT), 79.74 °C (PC-3) and 80.14 °C (PC-EF), respectively.

**Figure 3.**
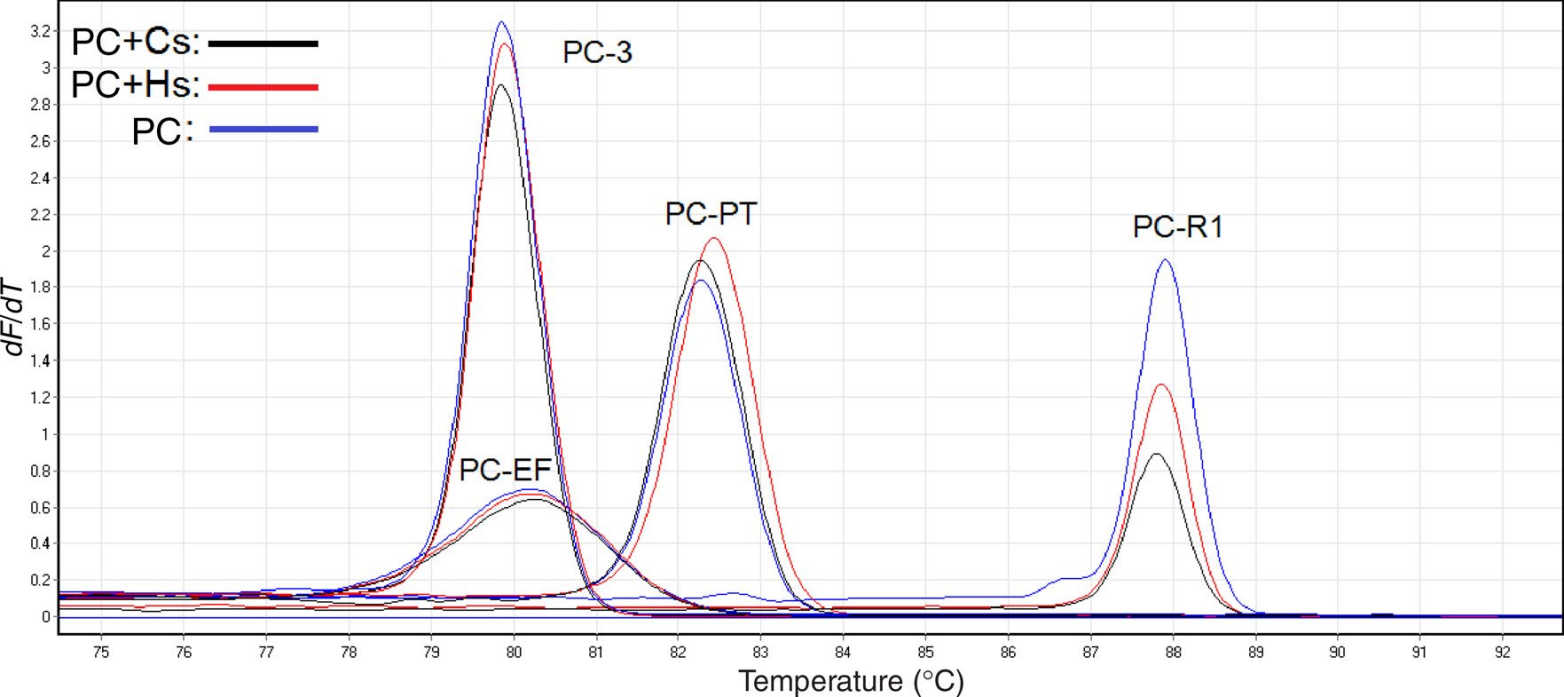
High-resolution melting (HRM) curves of *Psilocybe cubensis* in mixtures (mixed with *Cannabis sativa* or *Homo sapiens*) with four designed primers.

### Multiplex PCR HRM

Several chosen markers were amplified in a single multiplex PCR reaction, including five groups of PC-R1/PC-PT/PC-3, PC-R1/PC-3, PC-R1/PC-PT, PC-PT/PC-3 and PC-R1/PC-EF. As is shown in Supplementary Figure S5, all primer pairs within the same multiplex PCR reaction performed successful and independent melting curves, and the *T*_m_ values obtained in multiplex PCR HRM were consistent with the above data.

**Figure 7.**
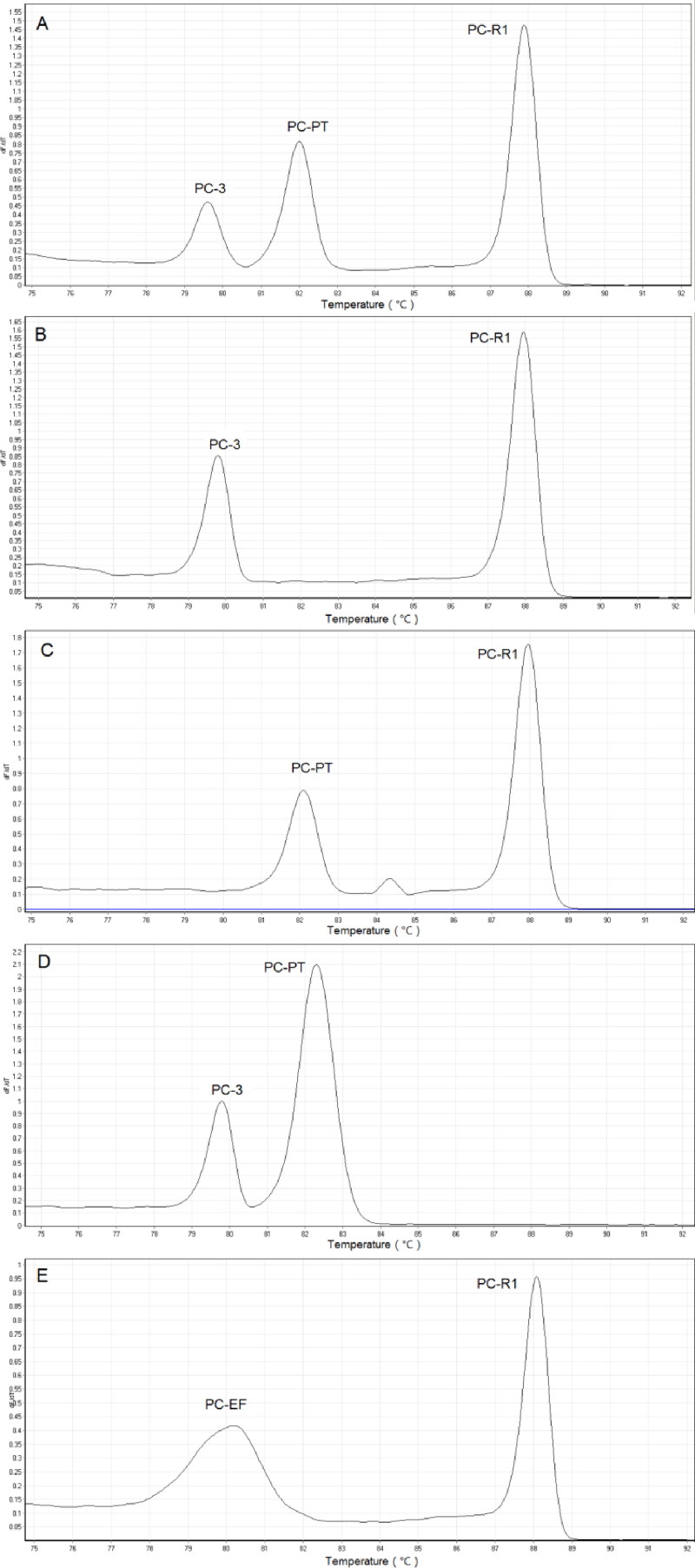
Multiplex PCR HRM assay using Eva Green™ for *Psilocybe cubensis*. (a) PC-R1 + PC-PT + PC-3, (b) PC-R1 + PC-3, (c) PC-R1 + PC-PT, (d) PC-3 + PC-PT and (e) PC-R1 + PC-EF.

## Discussion

Among the four chosen markers, glyceraldehyde 3-phosphate dehydrogenase and *tEF1α* gene were previously selected to target *P. cubensis* in Cowan and Elkins study [20], while the *RPB1* and *Psilocybin*-related phosphotransferase gene were firstly used for the identification of mushrooms. In our specificity study, four primer sets designed to amplify the four markers were tested over 22 species, including mushrooms, plants and human, and every primer pair appeared to different specificity. Among these primers, the primer set PC-3 amplifying glyceraldehyde 3-phosphate was the most specific, which only amplified five species including *P. cubensis*, while the PC-R1 amplifying *RPB1* was the least, which amplified *P. cubensis* and other 13 species. Furthermore, *P. cubensis* and *S. rugosoannulata* were both successfully amplified by all primer sets, which might be attributed to the fact that *P. cubensis* and *S. rugosoannulata* belong to the same family, Strophariaceae. Although the combination of four primer sets is not species-specific for *P. cubensis*, we could make a clear identification between these two species based on *T*_m_ values of PC-3 and PC-EF. These results demonstrated the ability of the primers, and the HRM analysis based on these primers might increase the resolution of present identification approaches for *P. cubensis*.

The sensitivity test was also performed by comparing the *dF*/*dT* values of melting curves obtained from different concentration of *P. cubensis*. For primer set PC-R1, PC-PT and PC-3, all DNA concentration produced complete melting curves even if 62.5 pg/µL of DNA generated relatively lower peaks. However, for primer set PC-EF, the *dF*/*dT* values of all concentration were obviously small, and 62.5 pg/µL produced a value of less than 0.5, suggesting that 62.5 pg/µL might be close to the lower limits of PC-EF (Figure 2). Similar trends were also observed in the reproducibility study. The low melting peaks of PC-EF may be attributed to the shorter length of its PCR fragments along with lower fluorescence binding affinity [22], but this shortcoming would become an advantage when assessing old and degraded samples. Taken together, the combination of four primer sets can be used for the detection of real-life samples in very low concentration.

In addition to sensitivity and repeatability assessment, the four primer sets were also analyzed on DNA mixtures containing *P. cubensis* and *H. sapiens* or *C. sativa* is one of the most common hallucinogens, and the *H. sapiens* DNA might be mixed with *P. cubensis* in gastric contents, which are both common samples in forensic drug-trafficking cases. In our mixture analysis, we used control sample of single *P. cubensis* as reference. No obvious divergence was observed between DNA mixtures and the control of single *P. cubensis*, neither was observed between two kinds of mixtures (Figure 3). These results were consistent with our specificity studies since no amplification was observed in *H. sapiens* and *C. sativa*. The results obtained from mixture testing suggested that these four primer sets analyzed using HRM technology can clearly detect DNA of *P. cubensis* in DNA mixes but cannot detect DNA of other species within the mixtures. Our mixture testing demonstrates the specificity of the primers for *P. cubensis* but also reveals the limitation of this method for the examination of other species contained by mixtures. At last, we sought to improve the efficiency of our identification method and therefore performed multiplex PCR HRM analysis on *P. cubensis*. All chosen primers mixed in a single multiplex PCR reaction generated distinct HRM curves and *T*_m_ values, indicating the feasibility of multiplex PCR HRM used for real-life samples.

The instrument of HRM was widely equipped in forensic laboratories, which is cost-effective and user-friendly for technicians [23–25]. The PCR HRM assay for the *P. cubensis* identification is also a reliable and effective strategy, which could serve as a confirmatory test for detecting the presence of *P. cubensis* DNA. In this study, four DNA markers of *P. cubensis* amplified by specific primer sets were analyzed by real-time PCR with the additional step of HRM assay, which clearly assessed the characteristics of target markers and discriminated the *P. cubensis* from other species. Furthermore, the results of validation studies confirmed the sensitivity, repeatability of these specific primers and their identification performance in mixtures. The significance of this method might be limited by the small sample sizes in our specificity study. The four primer sets were only tested across 22 species, which evokes the need for future study with more samples from different species to further evaluate the specificity of these primers. Additionally, this species-specific identification method appeared to limitations in mixtures for only *P. cubensis* could be detected, which demands complementary methods for the identification of *C. sativa* and other hallucinogens.

## Conclusion

In this study, we designed a combination of four primer sets and investigated a novel identification method specifically for *P. cubensis*. The HRM assay were used to analyze the amplicons of four primer sets, which were subsequently tested across several hallucinogenic mushrooms, including Psilocybe, Panaeolus and Gymnopilus, and some related mushrooms and plants. Based on this PCR HRM assay, we successfully discriminated *P. cubensis* from other species. Further, our mixture tests suggested that these four primer sets can clearly detect DNA of *P. cubensis* in DNA mixes but cannot detect DNA of other species within the mixtures. At last, our multiplex PCR assay indicated that different primer sets mixed in a single reaction could generate distinct HRM results, which could be applied for rapid examination of real-life samples.

The PCR HRM assay for the *P. cubensis* identification is a reliable and effective strategy, which could serve as a confirmatory test for detecting the presence of *P. cubensis* DNA. Our study provided an HRM-based approach for the confirmation of *P. cubensis*, which would be a powerful molecular tool in tracking distribution networks and criminal case evidences.

## Supplementary Material

Supplemental Material

## Notes

This study was supported by grants from the Shanghai Areas of Development for Society Planning Projects [grant number 19dz1200600], the National Natural Science Foundation of China [grant numbers 81930056 and 81625013], and the National Youth Talent Support Program [grant number WRQB2019].
