# Supplementary figures and images for: Multi-locus identification of Psilocybe cubensis by high-resolution melting (HRM)
Source: Forensic Sci Res. 2021 Apr 13;7(3):490–7. doi: 10.1080/20961790.2021.1875580 (PMC9639532; doi:10.1080/20961790.2021.1875580)

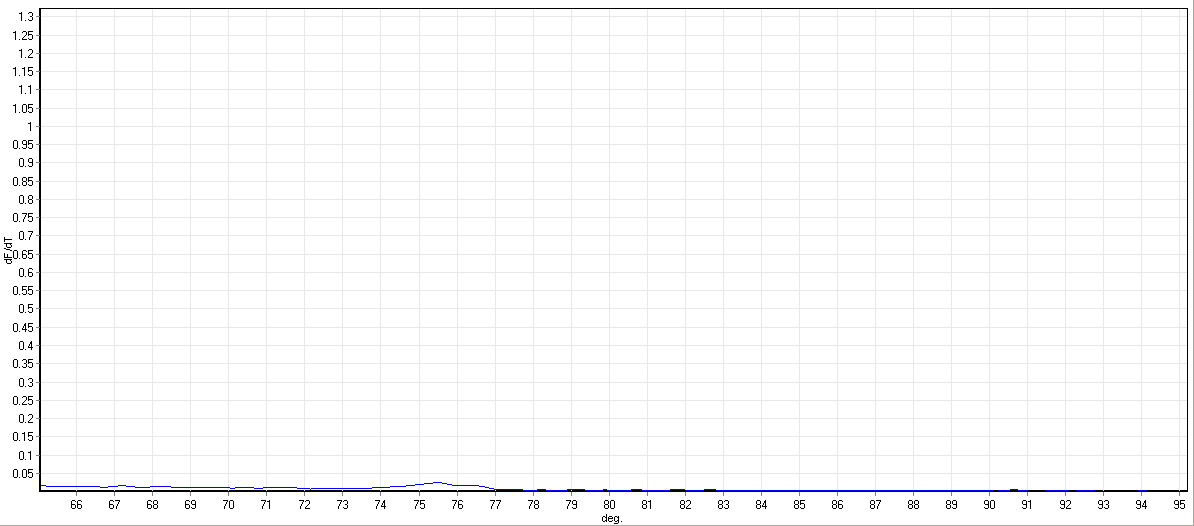

Supplement: Supplemental Material [file TFSR_A_1875580_SM5377.png]
